# Analysis of airway secretions in a model of sulfur dioxide induced chronic obstructive pulmonary disease (COPD)

**DOI:** 10.1186/1745-6673-1-12

**Published:** 2006-06-07

**Authors:** Ulrich Wagner, Petra Staats, Hans-Christoph Fehmann, Axel Fischer, Tobias Welte, David A Groneberg

**Affiliations:** 1 Department of Medicine, Pulmonary and Critical Care Division, Philipps-University, Baldingerstr., D-35043 Marburg, Germany; 2 Department of Medicine/Respiratory Medicine, Klinik Löwenstein, Geißhölzle 62, D-74245 Löwenstein, Germany; 3 Allergy-Centre-Charité, Otto-Heubner-Centre, Pneumology and Immunology, Charité – Universitätsmedizin Berlin, Free and Humboldt-University Berlin, Augustenburger Platz 1 OR-1, D-13353 Berlin, Germany; 4 Department of Respiratory Medicine, Hannover Medical School, Carl-Neuberg-Str. 1 OE 6870, D-30625 Hannover, Germany; 5 Institute of Occupational Medicine, Charité – Universitätsmedizin Berlin, Free and Humboldt-University Berlin, Ostpreussendamm 111, D-12207 Berlin, Germany

## Abstract

Hypersecretion and chronic phlegm are major symptoms of chronic obstructive pulmonary disease (COPD) but animal models of COPD with a defined functional hypersecretion have not been established so far. To identify an animal model of combined morphological signs of airway inflammation and functional hypersecretion, rats were continuously exposed to different levels of sulfur dioxide (SO2, 5 ppm, 10 ppm, 20 ppm, 40 ppm, 80 ppm) for 3 (short-term) or 20–25 (long-term) days. Histology revealed a dose-dependent increase in edema formation and inflammatory cell infiltration in short-term-exposed animals. The submucosal edema was replaced by fibrosis after long-term-exposure. The basal secretory activity was only significantly increased in the 20 ppm group. Also, stimulated secretion was significantly increased only after exposure to 20 ppm. BrdU-assays and AgNOR-analysis demonstrated cellular metaplasia and glandular hypertrophy rather than hyperplasia as the underlying morphological correlate of the hypersecretion.

In summary, SO2-exposure can lead to characteristic airway remodeling and changes in mucus secretion in rats. As only long-term exposure to 20 ppm leads to a combination of hypersecretion and airway inflammation, only this mode of exposure should be used to mimic human COPD. Concentrations less or higher than 20 ppm or short term exposure do not induce the respiratory symptom of hypersecretion. The present model may be used to characterize the effects of new compounds on mucus secretion in the background of experimental COPD.

## Introduction

Chronic obstructive pulmonary disease (COPD) is a chronic inflammatory airway disease that is characterized by hypersecretion, cough and inflammatory cell influx [1,2]. COPD is currently estimated to become the 3^rd ^most common cause of death in 2020 [3]. The clinical and morphological features of the disease are mediated by humoral [4] and/or neuronal mediators [5,6] and display protective first-line responses against inhaled irritants. However, within chronic exposure to noxious substances such as tobacco smoke, urban dust, or occupational factors [7-9], the originally protective mechanisms lead to a state of chronic phlegm production [10], airway inflammation, and cough, significantly limiting the quality of life [11]. Under these conditions therapeutic options such as the efficacy of topically administered drugs become negatively influenced and respiratory pharmacotransporters may be negatively influenced [12-15].

Next to tobacco smoke exposure, either active or passive, a number of other environmental factors can be associated to the development of COPD [16]. Sulfur dioxide belongs to these factors and its associations to airway diseases have been investigated since the London smogs [17], which are known to be one of the first characterized dramatic urban environmental exposures to air pollutants [18].

The WHO definition of COPD is only based on clinical criteria. Moreover, there are some morphological methods such as the Reid index to define histological features of the disease [19]. However, functional parameters, as found for the description of allergic obstructive airway diseases such as allergic bronchial asthma [20], do not exist. In this respect, in striking contrast to numerous existing experimental approaches to determine the inflammation in airway disease [21,22], models to characterize the secretory activity of the airway under the conditions of chronic airway inflammation have not been established so far. With regard to a situation of a lack in valid strategies for hypersecretion therapy there is an urgent need for novel established models to test new therapeutic options [23,24].

Next to chronic chough, that has only been recently addressed in greater detail by international research efforts [25-36], also mucus secretion belongs to the few respiratory symptoms with extremely limited therapeutic options. A variety of morphological approaches to characterize mucus secretion in the airways have been developed in the past years [37]. With the first identification of mucin proteins as the molecular backbone of airway mucus [38], morphological studies were able to determine mucin expression in respiratory cells of the upper [39] and lower airways [40-42]. Also, the pathogenesis of mucus cell metaplasia was assessed in a murine asthma model using morphometry [43]. However, these approaches can be efficiently used to determine the quality of the secreted mucus but not to exactly assess the secretory activity under basal or chronic inflammatory conditions as found in asthma or COPD. Therefore, an assay needs be established which encompasses a model of COPD with a defined hypersecretory state of the airways. The present study aimed to approach this target by combining an animal model of sulfur dioxide-induced lung lesions with the assessment of airway secretion by basal and stimulated secretion analysis. Different concentrations of SO2 were used to identify a model which encompasses both hypersecretion as defined by elevated basal secretion and stimulated secretion and morphological changes similar to those found in human COPD. Using this approach the first model displaying a clear association between hypersecretion as the prominent clinical symptom of COPD and the pathomorphological features of the disease should be established.

## Methods

### Animals

In total, 96 pathogen-free male Sprague-Dawley rats (Institut für Versuchstierzucht, Hannover, Germany), weighing 350–400 g were used. The animals were housed under standard laboratory conditions and fed *ad *libitum in accordance to the rules of the animal welfare act and the local animal committee.

### Exposure

For exposure to SO2, 4 animals were transferred to a plexi-glas chamber with a volume of 57.2 liters. Then, continuous exposure over 24 h to SO2 was started by a controlled dilution of air and SO2 from a reservoir containing SO2 in a concentration of 1000 mg/m3 using two parallel flow meters. 12 liter/min of the resulting different SO2-air mixtures were then directed through the plexi glass chamber using a third flow meter. A ventilator inside the chamber provided constant air movement and the atmosphere was measured using a hygrometer, thermometer and barometer. SO2 concentrations were measured online (ECS 102-1, MPSensor Symytems GmbH, Munich, Germany). Control groups were exposed in a similar plexi glass chamber to fresh air 12 liter/min instead of SO2 using the same conditions and measurement of SO2 concentration.

### Experimental design

**Figure 1:**
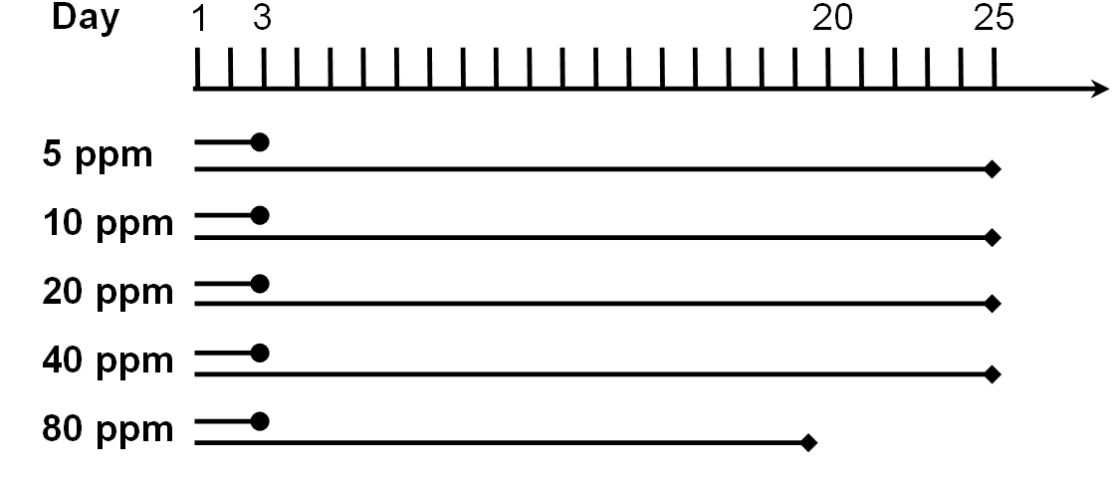
Exposure protocols.

The time-course and dose-relationship of possible changes in secretory activity was assessed. Therefore, different time and dose schedules were used: short-term (3 d) and long-term (20 – 25 d) periods of permanent exposure to SO2 were chosen to determine secretion under short and long term exposure (Figure 1).

Also, different doses of SO2 were examined and 6 groups for each period were assessed: 5 ppm, 10 ppm, 20 ppm, 40 ppm, 80 ppm and control (n = 4 in each experiment).

At the end of the assigned time-period airways were collected for direct measurement of secretory activity. In addition, tissues were perfusion fixed to study tissue remodeling by histology.

### Measurement of secretory activity

Airway secretory activity was measured as described in previously published studies [44-47]. In brief, a midline collar incision and median sternotomy were performed and the tracheas were excised and immediately transferred to 4°C cold M199 culture medium (Serva, Heidelberg, Germany). Connective tissue was removed and the tracheas were opened through a paries membranaceus-incision. The trachea preparations were then mounted in the chambers to obtain a mucosal and submucosal side, to which 7 ml of M-199 medium in Earl's balanced salt solution (equilibrated with carbogen at 37°C, pH 7.4) was added. Then, 50 μCi of Na_2_^35^SO_4 _was placed in the submucosal side of the preparation and allowed to equilibrate. The luminal solution was collected every 15 min and replaced by unlabeled fresh medium. After basal activity was measured, the stimulated secretion was assessed using acetylcholine. A luminal stimulation with 1 mmol/l was performed.

The collected probes were stored in cellulose dialysis tubings (cut-off mass 12000–14000 molecular mass) and dialyzed against distilled water containing unlabeled SO_4 _to replace non-incorporated Na_2_^35^SO_4_. To prevent bacterial degradation, sodium azide was added in a concentration of 10 mg/dl. The levels of radioactivity were then measured in a liquid scintillation counter with the counts of labeled macromolecules, which were previously characterized as representing the secretory activity rate of mucus [48-50].

### Histology

Histology was carried out on formalin-fixed lung specimen. 5 μm sections were cut from paraffin-embedded tissues and mounted on glass slides. Routine histology was then carried out as described previously [51] using hematoxylin-eosin, alcian blue and the Periodic-Acid-Schiff (PAS) reaction.

### Proliferative activity of glandular and surface epithelial cells

#### In vivo Bromodeoxyuridine (BrdU)-assay

An in vivo BrdU assay (each group n = 5) was performed as described previously [52]. One hour prior to termination of exposure, a single injection of 325 μl of 0.17 mM PBS-buffered BrdU (Boehringer, Mannheim, Germany) was administered intraperitoneally Sections were processed as described earlier and endogenous peroxidase activity inhibited by incubation with 0.5% (v/v) hydrogen peroxide in methanol for 30 min at room temperature. Subsequently, slices were digested with 0,004% pepsin solubilized in 0,1 n HCl and washed in PBS afterwards. Nuclease-digestion was performed with 10 U/ml EXO III (Boehringer, Mannheim, Germany) solubilized in 50 mM Tris-HCl at pH 8,0 as well as 10 mM di-mercapto-ethanol (Merck, Darmstadt, Germany) and 5% BSA. BrdU was detected immunohistochemically using a mouse anti BrdU antibody (Boehringer). This antibody was diluted 1:20 in 50 mM Tris-HCl, 7 mM MgCl_2_, 50 mM NaCl, 7 mM di-mercapto-ethanol and 1% (W/V) BSA. Incubation was performed at 37°C for 45 min. Rabbit anti-mouse Ig, diluted at 1:40 in 0,3 mM PBS was used as a link antibody, and finally mouse-PAP complex was added at 1:200 dilution. Peroxidase activitiy was detected with 0,01% (v/v) hydrogen peroxide and 0,05% (w/v) diaminobenzidine tetrahydrochloride (Dako, Hamburg, Germany). In tissue sections at least 500 cells were counted per specimen and tracheal region (superficial epithelium, glandular ducts and glandular acini).

#### Argyrophilic nucleolus organizer region (AgNOR)-analysis

To further assess cellular protein synthesis of glandular and epithelial cells, AgNOR analysis was performed [53]. AgNORs represent the two argyrophilic proteins nucleolin and nucleophosmin of the nucleolus organizer regions associated proteins, which are specifically attached to the nucleolus organizer regions and are directly involved in cellular protein synthesis. The sections were postfixed for 30 min in a 3:1 ethanol/acetic acid solution at room temperature. A silver colloid soution (1% gelatin and 2% formic acid) was mixedwith 50% siver nitrate in a proportion of 1:2 to obtain the final working solution for staining of AgNORs. The silver reaction carried out for 22–24 min in the dark. The sections were mounted in DPX without conterstain. The AgNOR analysis was performed by counting the number of AgNOR (black dots) per cell at a magnification of 1000× (each group n = 5 animals).

### Analysis of data

The secretory basal and stimulated activity is measured as counts per minute (cpm). Data are presented as ± S.D. Statistical analysis was performed using the ANOVA Friedman test and the Mann Whitney Wilcoxon U-test.

## Results

### Secretory activity

To determine the secretory activity in experimental bronchitis and to identify a model with proximity to human conditions concerning hypersecretion and morphological changes, animals were exposed to five different concentrations of SO2 and significant differences were found between the exposure and control groups.

### Short-term exposure to SO2

#### Basal secretory activity

**Figure 2:**
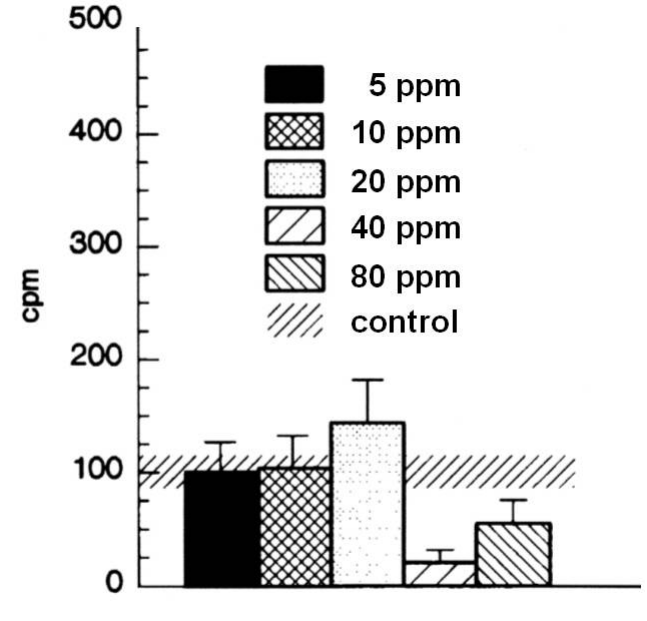
Secretory basal activity after 3 days of exposure. The tracheal secretory basal activity of 6 groups exposed to either 5, 10, 20, 40, 80 ppm SO2 or fresh air was assessed using a modified Ussing Chamber. Results are presented as mean ± SD cpm.

After three days of exposure the basal secretion for 5 ppm (100 ± 26.2 cpm (S.D.)), 10 ppm (103.6 ± 28.5 cpm), and 20 ppm (143.8 ± 38.3 cpm) did not differ significantly from the control group (85.7 ± 32.9 cpm). After exposure to 40 and 80 ppm, there was a nonsignificant tendency of decreased secretory activity (Figure 2).

#### Stimulated secretory activity

**Figure 3:**
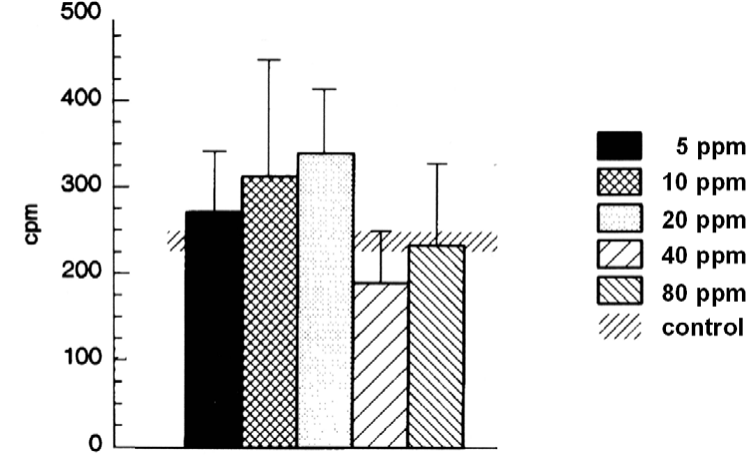
Stimulated secretory activity after 3 days of exposure. The acetylcholine-stimulated secretory activity of rats (for each group n = 4) exposed to either 5, 10, 20, 40, 80 ppm SO2 or fresh air as assessed by the modified Ussing Chamber technique. Results are presented as mean ± SD cpm.

The control group of animals which were only exposed to air in the chamber exhibited an acetylcholine-stimulated secretory activity of 224.6 ± 25.7 cpm which differed significantly from the basal secretory activity indicating the reactivity of the glands to parasympathetic stimulation. In contrast to these differences, no significant changes were present within the 5 ppm, 10 ppm, 20 ppm, 40 ppm and 80 ppm groups when compared to the control group (Figure 3).

### Long-term exposure to SO2

#### Basal secretory activity

**Figure 4:**
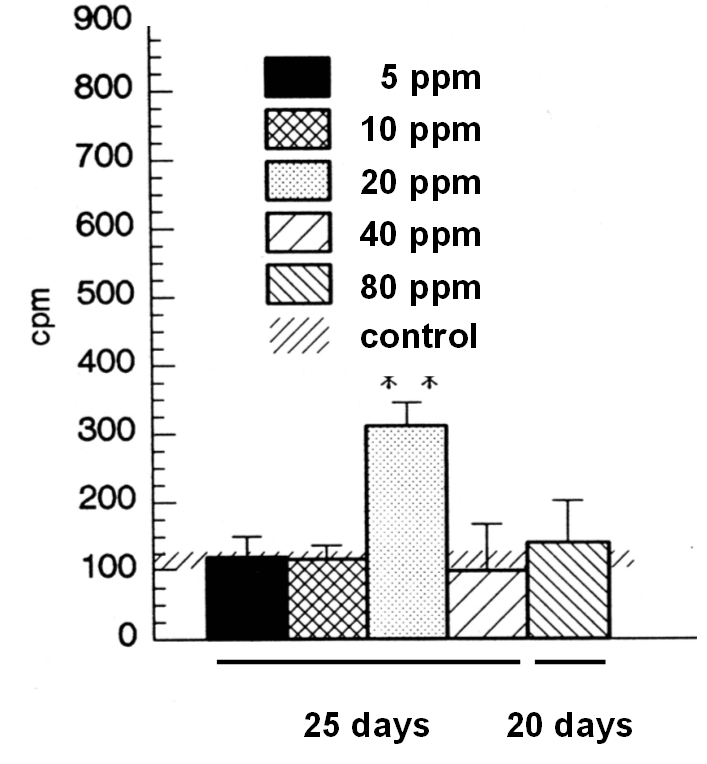
Secretory basal activity after 25 days of exposure to 5, 10, 20, 40 ppm SO2 or fresh air and 20 days exposure to 80 ppm SO2. Results are presented as mean ± SD cpm. ** indicates p < 0.01.

After 25 days of exposure to SO2 in concentrations of 5 ppm, 10 ppm and 20 ppm, there were no significant differences found for 5 ppm and 10 ppm basal secretion. In contrast, there was a highly significant increase present in the 20 ppm group with a secretory activity of 309.1 ± 36.9 cpm vs. 99.5 ± 27.1 cpm (control, *p *< 0.01). In higher concentrations, no significant differences were found. In this respect, in the 80 ppm group which was exposed for 20 days, the basal secretion was 140.1 ± 64.6 cpm (Figure 4).

#### Stimulated secretory activity

**Figure 5:**
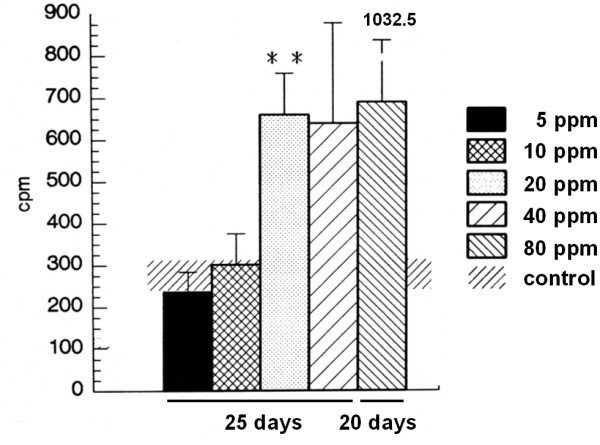
Stimulated secretory activity after 25 days of exposure to 5, 10, 20, 40 ppm SO2 or fresh air and 20 days exposure to 80 ppm SO2. The acetylcholine-stimulated secretory activity of rat tracheas (for each group n = 4) was assessed by the modified Ussing Chamber technique. Results are presented as mean ± SD cpm. ** indicates p < 0.01

The groups of animals which were exposed for 25 days to 5 ppm or 10 ppm did not differ significantly from the control group concerning acetylcholine-stimulated secretion. In contrast, there was a highly significant increase in the 20 ppm group (658.7 ± 99.1 cpm vs. 242.5 ± 73.4 cpm). Also, values for the 40 ppm group were increased. The stimulated secretory activity for 80 ppm was recorded on day 20 as further exposure to SO2 was lethal and a value of 695 ± 337.5 cpm was found (Figure 5).

### Airway remodeling

To correlate the hypersecretion as assessed by basal and acetylcholine-stimulated secretory activity to changes in the airway structure in order to identify a model which exhibits both typical morphological features of COPD and the predominant clinical feature of the disease, hypersecretion, the airways of all 12 groups were examined for airway remodeling and differences were found.

#### Control group

**Figure 6:**
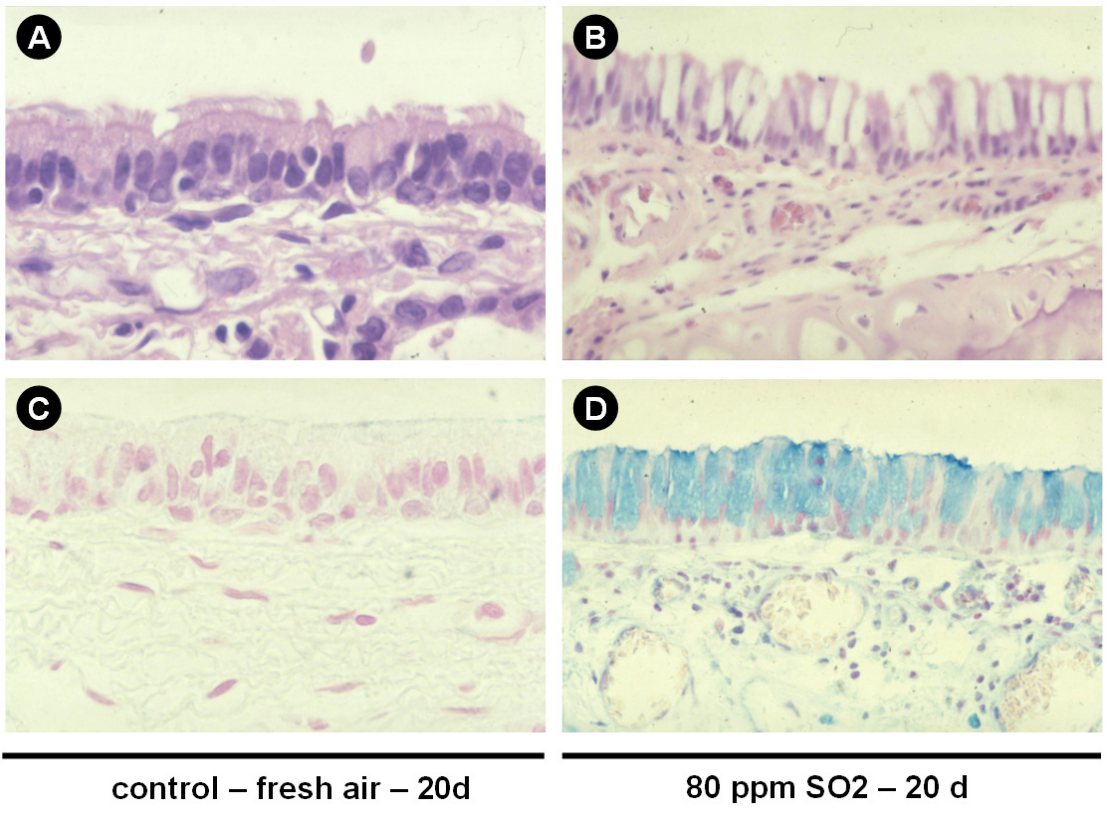
Morphological changes. Representative figures for control animals (A, HE staining; C, Alcian blue) and animals exposed to 80 ppm SO2 for 20 days (B, HE staining; D, Alcian blue) illustrate airway remodeling with organized, histologically normal epithelium in the control group and structurally altered epithelium after 20 days of exposure. Epithelial cells displayed an increased height and a loss of ciliae. Alcian blue staining (C, D) demonstrates an increase of mucus positive cells within the epithelium and a loss of ciliae. Original magnification ×400.

In control tissues, the surface epithelium was characterized by typical ciliated epithelial cells and an intact basal membrane and very few mucus cells. Mucus glands were found in the submucosal layer between cartilaginous tissues (Figure 6).

#### Short-term exposure to SO2

In the surface epithelium, a concentration of 5 ppm did not influence either the ciliae or shape of the epithelial cells. From a concentration of 10 ppm single cell necrosis and an increasing loss of ciliae were found which increased at higher concentrations. At 80 ppm, the structure of the surface epithelium is disintegrated with partial denudation of the basal membrane and inflammatory cell influx.

In the submucosal layer, from 5 ppm increasing to higher concentrations, inflammatory infiltrates and progredient edema and vascular congestion were found. The infiltrations also reach the submucosal glands.

#### Long-term exposure to SO2

In contrast to the morphology of short-term exposed airways, after 20 and 25 days of exposure the surface epithelium appeared atypically reorganized with an increased height of the epithelial cells and shortened/decreased ciliae. Also, 20, 40 and 80 ppm exposure led to an increase of mucus secreting PAS-positive cells (Figure 6).

### Proliferative activity of glandular and surface epithelial cells

**Figure 7:**
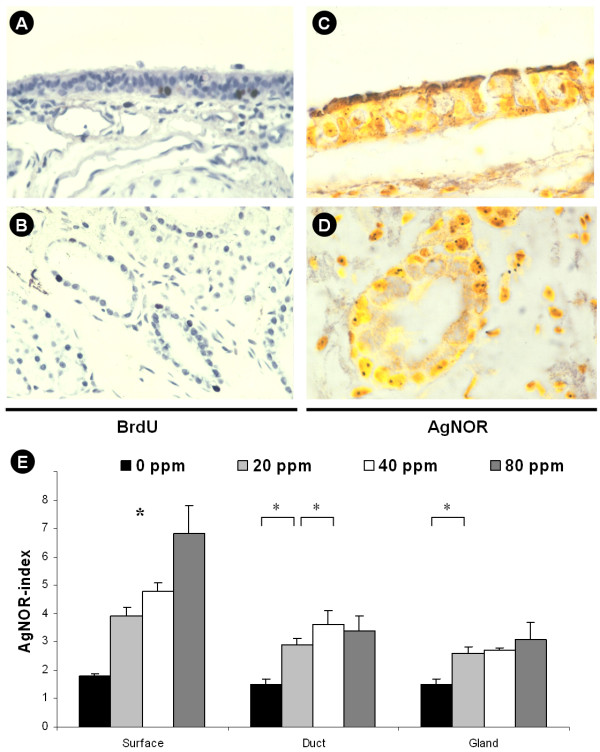
Proliferative activity of glandular and surface epithelial cells. To assess the proliferative activity, an in vivo BrdU-assay and AgNOR-analysis were performed. Positive staining indicating BrdU incorporation was only found occasionally in the surface epithelium and in glands (A, B). In contrast, AgNOR-analysis demonstrated argyrophilic nucleolar organizer regions (C, D) and the AgNOR index (E) revealed significant differences (*, p < 0.05) between exposed and nonexposed groups. Original magnification ×400 (×1000, D).

To characterize the proliferative activity of glandular and surface epithelial cells, an in vivo BrdU-assay was performed. However, in contrast to proliferative activity of control organs (esophagus), no significant staining for BrdU was found for glandular structures and surface epithelium and positive staining was only found occasionally (Figure 7).

Also, AgNOR-analysis was performed (Figure 7). Here, significant differences between the groups were found in the long term-exposed animals. In the glands, the only significant differences were present between the 20 ppm group and the control group (p < 0.05). In the area of the surface epithelium, all groups differed significantly from each other. I.e. an index of 1.8 ± 0.1 was found for control animals and 3.9 ± 0.3 (p < 0.05). For ductal epithelial cells, significant differences were only found between control and 10 ppm and 10 and 20 ppm (p < 0.05).

## Discussion

Airway mucus protects the respiratory tract against noxious substances und physiological conditions [54,55]. However, under pathological conditions such as COPD, hypersecretion may negatively influence quality of life and belongs to the key features of the disease [56].

In contrast to the large number of animal models and techniques to assess pathophysiological mechanisms occurring in allergic bronchial asthma [43], only a few animal models of COPD have been established so far. These models use exposure to either tobacco smoke [57], nitrogen dioxide [58] or sulfur dioxide [59] and all produce features which mimic COPD morphologically with inflammatory changes and variable emphysema. The important clinical feature of hypersecretion, which is a keystone of COPD, has not been assessed directly in animal models so far and our initial observations led us to conclude that process parameters and exposure periods could dramatically affect the production of mucus in these models. Therefore, hypersecretion may not be an integrative feature of previously described models of COPD and thus, the integrity of these models as an approach to mimic human COPD may be questioned. To unravel these questions, the present study aimed to establish a model of COPD which does not only display morphological changes but also a functional hypersecretion as found in patients with the disease. An assay should be identified which allows the assessment of the secretory activity under experimental conditions of COPD.

To functionally assess airway secretory activity, previously established techniques were applied which allow to measure both the basal and the metacholine-stimulated tracheal secretion [44-46,48-50].

As exposure to sulfur dioxide has previously been reported to lead to an experimental form of COPD [59], the present study aimed to identify a mode of SO2 exposure which encompasses not only morphological features of COPD, but also a state of hypersecretion and therefore allows to study the effects of potential new secretion-modulating compounds in the background of airway inflammation and hypersecretion in future. Five different concentrations of SO2 were used in two periods. Routine histopathology revealed signs of bronchitis with increasing concentrations. Within the long-term groups of exposure, signs of fibrotic airway remodeling were found. Similar changes have also been reported in other studies using SO2 as noxious compound to evoke experimental bronchitis [59]. The aim was to correlate morphological changes in the rat airways to hypersecretion and to find a protocol of exposure which ensures that both characteristic features of bronchitis and hypersecretion are present and can be assessed.

Basal and acetylcholine-stimulated secretory activities were assessed to provide two different scores of mucus secretion. Hypersecretion was not found in every mode of SO2 exposure even in the presence of morphological signs of bronchitis, indicating that previously reported protocols and models may lack a functional hypersecretion and therefore be biased by not displaying a key feature of COPD. In this respect, morphological analysis of the three day exposure groups demonstrated that changes in the airway wall structure can be found after this short period of exposure with the tissues exhibiting signs of acute edema, loss of ciliar integrity and inflammatory cell influx with increasing concentrations of SO2. However, basal and stimulated secretory activity did not reveal any sign of hypersecretion and instead, in the group of 40 and 80 ppm exposure, tendencies for decreases in the secretion were found, indicating that short term exposure to a range of 5 to 80 ppm SO2 can not lead to an adequate model of bronchitis encompassing both inflammatory and hypersecretory changes.

In the groups which were exposed to SO2 for 25 (20) days, a highly significant increase was present in the basal secretory state of the 20 ppm group with 309.1 ± 36.9 cpm vs. 99.5 ± 27.1 cpm in the control group, and also the stimulated secretion was elevated in this group. These functional changes were concomitant with morphological changes of airway remodeling and chronic inflammation including fibrosis. In contrast, higher concentrations did not seem to increase basal or stimulated secretion more significantly and concentrations higher than 80 ppm were not tolerated by the animals.

To characterize the proliferative activity of glandular and surface epithelial cells, *in vivo *BrdU assays and AgNOR-analysis were performed. However, in contrast to proliferative activity of control organs (esophagus), no significant staining for BrdU was found for glandular structures and surface epithelium. As BrdU is a nucleoside analogue that is incorporated in the place of thymidine when cells divide [60], the absence of significant BrdU staining indicates that the observed hypersecretion is not due to an increased replicative chromosomal activity of glandular cells. Therefore, an AgNOR-analysis was performed which is related to rRNA transcriptional activity and cellular protein biosynthesis [53] and a significant difference was found between the 20 ppm group and the control group but not for higher or lower concentrations.

The dramatic morphologic changes especially of the superficial epithelium without significant mitotic activity and without remarkable BRDU incorporation suggests that a metaplasia with subsequent hypertrophy of the glandular apparatus rather than hyperplasia as the underlying morphological correlate of the hypersecretion. These findings demonstrate that the exposure of Sprague-Dawley rats over 25 days to a concentration of 20 ppm SO2 leads to a model of experimental COPD with mucus cell metaplasia and glandular hypertrophy, which encompasses both characteristic clinical and morphological features of the disease. The possibility to functionally assess the secretory activity under defined experimental conditions offers the chance to study the effects of potential new compounds on airway secretion in COPD. Also, the effects of mediators of inflammation including reactive oxygen species [61] or neuromediators [62,63] and drug transport mechanisms [64,65] can be assessed in a setting of phlegm production and bronchitis.

As it has been shown that the eradication of inflammatory cells does not automatically lead to an improvement of clinical symptoms in airway diseases such as allergic asthma [66], other features of airway diseases such as hypersecretion or cough are currently focused as a base for new therapeutic options to improve the quality of life. In the light of these new strategies [67], models to assess cough and hypersecretion are required [68]. Therefore, the presently identified model of 25 day exposure with 20 ppm SO2 may find a broad application. However, SO2 is a relatively harmful gas and its toxicity may therefore limit a wide-spread use [69]. Currently, there are a number of other COPD models available [24]. These include the exposure to tobacco smoke and future studies on the secretory state in different protocols of tobacco smoke concentration and time exposure may offer an alternative to the presently established protocol.

In conclusion, the present study assessed the influence of five different concentrations of SO2 in two periods of exposure. Part of the presently used concentrations which are much higher than international threshold values did not lead to any changes. Among the different concentrations it was found that only a 25 days lasting period of exposure to 20 ppm of SO2 leads to a experimental model of COPD which does not only show morphological signs of the disease but also included the predominant clinical feature of mucus hypersecretion. The model may be used in future to determine the role of mediators and effects of new aerosolized compounds on airway secretion in the background of COPD.

## Authors' contributions

UW, PS, AF, HCF, TW and DAG have been involved in the design and conduct of the study. Also they have participated in drafting the article or revising it critically for important intellectual content. They have all given approval of the study to be published

## Declaration of competing interests

The author(s) declare that they have no competing interests.
